# Correlation of neutrophil-to-lymphocyte ratio and platelet-to-lymphocyte ratio with serum α-klotho levels in US middle-aged and older individuals: Results from NHANES 2007–2016

**DOI:** 10.1016/j.pmedr.2024.102877

**Published:** 2024-09-06

**Authors:** Rui Du, Jie Liu, Xiaoyan Tang, Zili Chen, Lei Guan, WenHong Gao, Wei Huang

**Affiliations:** a Department of Ultrasound, General Hospital of Central Theater Command, No.627, Wuluo Road, Wuhan 430070, Hubei, China; b Department of Ultrasound, International Mongolian Hospital of Inner Mongolia, No. 83, University East Street, Hohhot 010020, Inner Mongolia, China; c Department of Cardiology, General Hospital of Central Theater Command, No.627, Wuluo Road, Wuhan 430070, Hubei, China

**Keywords:** NHANES, α-Klotho, Aging, NLR, PLR, Systemic inflammation, Cross-sectional study

## Abstract

**Background:**

The α-klotho (αKl) is widely accepted as an anti-aging and anti-inflammatory protein. However, it is rarely reported on the function and mechanism of αKl in the overall population (including healthy people and those with history of chronic disease). The neutrophil-to-lymphocyte ratio (NLR) and platelet-to-lymphocyte ratio (PLR) are established as predictors of systemic inflammation. This study aims to investigate the relationship between NLR, PLR, and αKl levels in the overall population.

**Methods:**

Data from 10,124 adults aged 40 years old and above, collected from NHANES 2007–2016, were analyzed. Associations between NLR, PLR, and αKl levels were assessed by weighted multivariate linear regression analyses, adjusting for potential confounders. Subgroup analysis was conducted by gender, age, diabetes, cardiovascular disease, and chronic kidney disease.

**Results:**

Weighted linear regression models showed that a significant negative correlation was observed between both NLR and PLR with αKl levels. Subgroup analysis revealed that the negative correlation between NLR and serum αKl levels was not significant in individuals aged 40–59 years and males, while this relationship remained stable across most other subgroups. The negative correlation between PLR and serum αKl levels was consistent across most subgroups but not significant in individuals with cardiovascular disease.

**Conclusion:**

Our study revealed a significant negative relationship between inflammatory markers (NLR and PLR) and serum αKl levels, suggesting systemic inflammation may be linked to reduced αKl expression. Subgroup analyses showed that the relationship varies across different demographic and health-related factors. We provided insight into the significance of managing inflammation and preserving αKl levels.

## Highlights

• The study selected a large, representative sample from five consecutive NHANES cycles, ensuring robust and generalizable results. Systemic inflammation markers (neutrophil-to-lymphocyte ratio and platelet-to-lymphocyte ratio) were found to be significantly associated with lower serum α-klotho levels.
• The relationship between neutrophil-to-lymphocyte ratio, platelet-to-lymphocyte ratio, and α-klotho varies by age, gender, and the presence of chronic diseases such as hypertension, diabetes, cardiovascular disease, and chronic kidney disease. Elevated neutrophil-to-lymphocyte ratio and platelet-to-lymphocyte ratio may contribute to reduced α-klotho expression, potentially accelerating the aging process.
• These findings highlight the possibility of systemic inflammation inhibition in preserving α-klotho levels, which may be supposed as a novel therapy in age-related disorders.

## 1 Introduction

The α-klotho (αKl) is widely accepted as an anti-aging and anti-inflammatory protein (Kuro-o et al., 1997). A subset of this protein, αKl, is encoded by the klotho gene and predominantly manifests as a transmembrane protein in the kidney (Bergmark et al., 2019). αKl is reported to have antioxidative processes and is believed to play a crucial hormone-like role with anti-inflammatory properties. The neutrophil-to-lymphocyte ratio (NLR) and platelet-to-lymphocyte ratio (PLR) are well-established markers of systemic inflammation and have been extensively studied in relation to various diseases due to their easy availability and high sensitivity (Gutta et al., 2022, Song et al., 2024, Xu et al., 2024). Elevated NLR and PLR have been associated with poorer outcomes in cardiovascular diseases (CVD) (Garcia-Escobar et al., 2023), chronic kidney disease (CKD) (Yuan et al., 2019), cancers (Cupp et al., 2020, Nost et al., 2021), and other inflammatory conditions. Meanwhile, previous research suggests that systemic inflammation may negatively impact αKl expression (Zhao et al., 2024). Differential expression of αKl has been noted across various inflammatory-related diseases, such as CVD (Donato et al., 2018), CKD (Neyra et al., 2020), diabetes (Hua et al., 2021), and some cancers (Ligumsky et al., 2022). The association between inflammation and αKl has been investigated in the context of CVD, CKD, and rheumatoid arthritis (Martin-Nunez et al., 2020, Wu et al., 2019, Zhao et al., 2024). However, the relationship between NLR, PLR, and αKl level remains unclear, particularly in a large and diverse population (the overall population). Thus, this study aims to explore the relationships in adults using data from the 2007–2016 National Health and Nutrition Examination Survey (NHANES).

## 2 Materials and methods

### 2.1 Study design and participants

The data utilized in this study were obtained from the NHANES, a program conducted by the National Center for Health Statistics (NCHS) under the Centers for Disease Control and Prevention (CDC). NHANES is a continuous, cross-sectional survey designed to assess the health and nutritional status of adults and children in the U.S. civilian, non-institutionalized population. The survey employs a complex, multistage probability sampling design to ensure representative sampling. The NCHS Ethics Review Board approved the original research, with all participants providing written informed consent. According to the Ethics Review Board of the General Hospital of Central Theater Command, our study was based on publicly available anonymized databases and has been exempted from the ethical review.

NHANES collects a wide range of data through interviews, physical examinations, and laboratory tests. This survey includes demographic, socioeconomic, dietary, and health-related information. Laboratory tests conducted in NHANES include blood and urine analyses, which provide valuable biochemical data. For this study, we specifically utilized data from the NHANES cycles spanning 2007 to 2016, focusing on complete blood count (CBC) measurements and serum α-klotho (SαKl) levels. This period was selected because SαKl were only conducted during these years for participants aged 40–79.

**Fig. 1:**
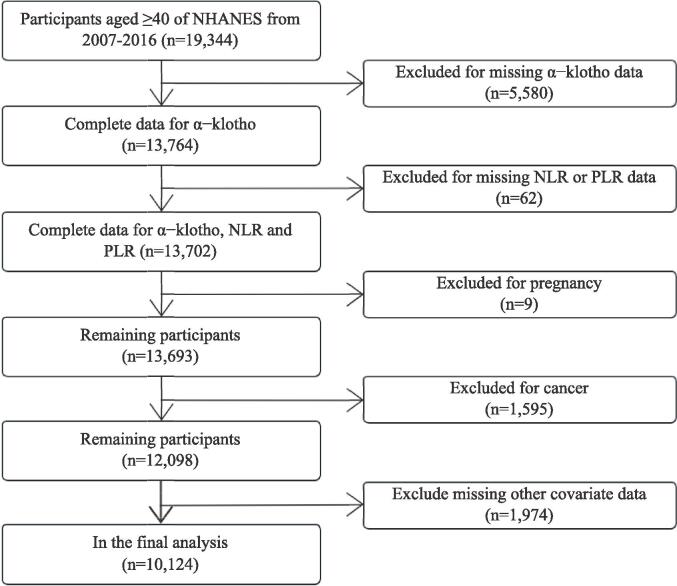
Flowchart of participant selection for adults aged 40 and above from NHANES 2007–2016. NLR, Neutrophil-to-lymphocyte ratio; PLR, Platelet-to-lymphocyte ratio.

Participants included in the analysis were those aged 40 years and older with available data on NLR, PLR, and SαKl levels. Individuals with missing data for any of these variables were excluded from the study. The primary outcome variable was the SαKl level, measured using enzyme-linked immunosorbent assay (ELISA) methods. The main predictor variables were NLR and PLR, calculated from complete blood count data. The sequence of participant interrogation and the flowchart of participant selection are depicted in Fig. 1.

### 2.2 NLR and PLR assessment

The NLR and PLR were derived from CBC data obtained during NHANES physical examinations. Blood specimens are processed, stored, and sent to the Division of Laboratory Sciences at the National Center for Environmental Health, CDC, for analysis. Blood samples were collected by trained phlebotomists following standardized protocols and analyzed in CDC-certified laboratories. The NLR was calculated by dividing the absolute neutrophil count by the absolute lymphocyte count. In contrast, the PLR was calculated by dividing the absolute platelet count by the absolute lymphocyte count. The accuracy and reliability of these measurements were ensured through rigorous quality control procedures in the NHANES laboratory methodology.

### 2.3 SαKl level

SαKl levels were measured using a highly sensitive and specific ELISA kit by Immuno-Biological Laboratories International, Japan. The αKl ELISA assay was conducted by trained laboratory personnel according to standardized protocols provided by the manufacturer (Hayrapetyan et al., 2023). Blood samples were collected from participants via venipuncture, processed, and stored at appropriate temperatures until analysis. Each sample was tested twice, and the mean value was determined. Quality control procedures, including calibration standards and control samples, were strictly followed to ensure the accuracy and precision of the measurements—the samples analyzed with intra- and inter-assay coefficients of variation typically less than 5 %. The mean SαKl level was 698.0 pg/mL, ranging from 285.8 to 1638.6 pg/mL (Yamazaki et al., 2010).

### 2.4 Covariate variables

Several covariate variables were included in our study to account for potential confounders and ensure the robustness of our findings. These variables were selected based on established criteria. They included age, gender (female or male), race/ethnicity (Mexican American, other Hispanic, non-Hispanic White, non-Hispanic Black, and others), and education level (less than high school, high school or general educational development, above high school). Body mass index (BMI) was classified according to World Health Organization criteria into normal weight (BMI < 25), overweight (BMI 25–30), and obese (BMI ≥ 30) (James, 2018). The family income to poverty (PIR) ratio was categorized into three groups: less than 1.30, 1.30–2.99, and 3.00 or higher. Smoking status was divided into never smokers (less than 100 cigarettes in a lifetime), former smokers (100 or more cigarettes but not currently smoking), and current smokers (100 or more cigarettes and currently smoking). Alcohol consumption was categorized based on previously published criteria into never drinkers, former drinkers, light-to-moderate drinkers, and heavy drinkers (Jiang et al., 2023). Physical activity (PA) levels were categorized as inactive, moderate, or vigorous. Diabetes was categorized based on previously published criteria (Du et al., 2023). Hypertension was defined by a systolic blood pressure of 140 mmHg or higher and/or diastolic blood pressure of 90 mmHg or higher or regular use of antihypertensive drugs. The identification of CVD and CKD relied upon a physician's diagnostic confirmation.

### 2.5 Statistical analysis

The analyses accounted for the complex design of the National Health Interview Survey by incorporating strata, primary sampling units, and sample weights. To compare baseline characteristics, weighted linear regression was used for continuous variables, and weighted chi-square tests were applied for categorical variables. The results for categorical variables were presented as weighted proportions with 95 % confidence intervals, while continuous variables were reported as weighted means ± standard error.

Multivariate linear regression analyses assessed the association between NLR, PLR, and SαKl levels. In accordance with the Strengthening the Reporting of Observational Studies in Epidemiology guidelines, we tested three models: Model 1 with no adjustment. Model 2 adjusted for age, gender, and race/ethnicity. Model 3 was further adjusted for BMI, marital status, education level, PIR, smoking status, alcohol consumption, physical activity, hypertension, diabetes, CVD, and CKD. Additionally, subgroup analyses were conducted based on gender, age (</≥ 60 years), hypertension, diabetes, CVD, and CKD. All statistical analyses were performed using R software (version 4.2.0) and Free Statistics software (versions 1.8). Statistical significance was defined as a *P* value < 0.05.

## 3 Results

### 3.1 Baseline characteristics

The dataset comprised 10,124 adults aged 40 years or older who participated in NHANES interviews from 2007 to 2016, with 51.35 % female. The weighted demographic characteristics and other covariates of the participants were presented based on gender (shown in Table 1). The mean age was 55.5 ± 0.2 years for females and 54.7 ± 0.2 years for males. The level of SαKl was higher in females than in males (867.92 ± 6.63 pg/mL *v.s* 828.90 ± 6.32 pg/mL, *P* < 0.001). Females showed a slightly lower NLR compared to males (2.16 ± 0.02 *v.s* 2.32 ± 0.02, *P* < 0.001), while the mean PLR was higher in females (131.99 ± 1.19 vs 123.67 ± 1.03, *P* < 0.001).

**Table 1:** Characteristics of adults aged 40 and above by gender from NHANES 2007–2016.

Characteristic	Total Weighted mean ± SE / weighted % (95 % CI)	Gender		*P*
		Female Weighted mean ± SE / weighted % (95 % CI)	Male Weighted mean ± SE / weighted % (95 % CI)	
**Age, years**	55.1 ± 0.2	55.5 ± 0.2	54.7 ± 0.2	<0.001
**α-klotho, pg/mL**	848.94 ± 5.38	867.92 ± 6.63	828.90 ± 6.32	<0.001
NLR	2.23 ± 0.02	2.16 ± 0.02	2.32 ± 0.02	<0.001
PLR	127.94 ± 0.94	131.99 ± 1.19	123.67 ± 1.03	<0.001
**Race/ethnicity, %**				<0.001
Mexican American	6.86 (5.45, 8.27)	6.52 (5.01, 8.04)	7.21 (5.52, 8.91)	
Other Hispanic	4.58 (3.57, 5.60)	4.56 (3.42, 5.69)	4.61 (3.48, 5.74)	
Non-Hispanic White	72.85 (64.85, 80.84)	72.75 (69.55, 75.96)	72.95 (69.79, 76.10)	
Non-Hispanic Black	9.45 (8.30, 10.59)	10.35 (8.57, 12.14)	8.49 (7.12, 9.86)	
Others	6.26 (5.44, 7.09)	5.81 (4.95, 6.68)	6.74 (5.67, 7.81)	
**BMI, %**				<0.001
Normal weight	23.98 (21.71, 26.24)	28.64 (26.74, 30.53)	19.06 (17.38, 20.74)	
Over weight	35.30 (32.21, 38.39)	29.96 (28.06, 31.86)	40.93 (38.89, 42.98)	
Obese	40.73 (37.63, 43.82)	41.41 (39.15, 43.66)	40.01 (37.92, 42.10)	
**Marital status, %**				<0.001
Married/living with partner	70.62 (64.68, 76.55)	65.38 (63.64, 67.12)	76.14 (74.04, 78.25)	
Living alone	29.38 (27.31, 31.46)	34.62 (32.88, 36.36)	23.86 (21.75, 25.96)	
**PIR, %**				0.02
Low	17.71 (15.94, 19.47)	18.47 (16.57, 20.36)	16.90 (15.00, 18.80)	
Middle	26.21 (23.73, 28.69)	26.89 (25.08, 28.69)	25.49 (23.47, 27.51)	
High	56.09 (50.55, 61.63)	54.65 (51.81, 57.48)	57.61 (54.52, 60.69)	
**Education level, %**				0.23
Less than high school	16.36 (14.66, 18.06)	15.81 (13.94, 17.67)	16.95 (15.03, 18.87)	
High school or general educational development	22.83 (20.38, 25.28)	22.49 (20.78, 24.20)	23.18 (21.32, 25.04)	
Above high school	60.81 (55.41, 66.20)	61.70 (59.01, 64.39)	59.87 (57.11, 62.62)	
**Physical activity, %**				<0.001
Inactive	49.35 (45.20, 53.50)	49.71 (46.87, 52.56)	48.97 (46.26, 51.68)	
Moderate	30.98 (27.75, 34.22)	33.63 (31.47, 35.79)	28.19 (25.76, 30.62)	
Vigorous	19.67 (17.21, 22.12)	16.66 (14.51, 18.81)	22.84 (20.47, 25.21)	
**Smoking status, %**				<0.001
Never	52.37 (48.48, 56.26)	57.96 (56.15, 59.77)	46.47 (44.17, 48.77)	
Former	28.89 (25.94, 31.84)	24.86 (22.82, 26.89)	33.15 (31.10, 35.19)	
Current	18.74 (17.00, 20.49)	17.18 (15.55, 18.82)	20.39 (18.74, 22.03)	
**Alcohol consumption, %**				<0.001
Never	10.39 (9.23, 11.54)	14.60 (13.04, 16.16)	5.93 (4.87, 7.00)	
Former	17.67 (16.14, 19.21)	16.92 (15.49, 18.36)	18.46 (17.08, 19.85)	
Light-to-moderate	55.66 (50.65, 60.67)	56.14 (53.47, 58.81)	55.15 (52.66, 57.64)	
Heavy	16.29 (14.76, 17.81)	12.34 (10.92, 13.76)	20.45 (18.65, 22.25)	
**Diabetes, %**				<0.001
No	70.90 (65.09, 76.72)	72.94 (71.34, 74.55)	68.75 (67.01, 70.49)	
Borderline	10.28 (9.16, 11.41)	9.22 (8.19, 10.26)	11.40 (10.29, 12.51)	
Yes	18.81 (17.42, 20.21)	17.83 (16.43, 19.23)	19.85 (18.20, 21.50)	
**Hypertension, %**				0.2
No	52.97 (48.65, 57.30)	53.80 (52.07, 55.52)	52.10 (50.09, 54.11)	
Yes	47.03 (43.53, 50.52)	46.20 (44.48, 47.93)	47.90 (45.89, 49.91)	
**CVD, %**				<0.001
No	89.83 (83.07, 96.60)	91.88 (91.02, 92.74)	87.67 (86.36, 88.99)	
Yes	10.17 (9.20, 11.13)	8.12 (7.26, 8.98)	12.33 (11.01, 13.64)	
**CKD, %**				0.01
No	84.66 (78.15, 91.18)	83.31 (81.80, 84.81)	86.10 (84.88, 87.32)	
Yes	15.34 (14.05, 16.63)	16.69 (15.19, 18.20)	13.90 (12.68, 15.12)	

Values are presented as weighted means ± standard errors (SE) or weighted frequencies (95 % confidence interval [CI]).

*P* value was determined by linear regression model for continuous variables and Chi-square test for categorical variables.

**Abbreviations:** NLR, Neutrophil-to-lymphocyte ratio; PLR, Platelet-to-lymphocyte ratio; BMI, body mass index; PIR, Ratio of family income to poverty; CVD, cardiovascular disease; CKD, chronic kidney disease.

In addition, more males belonged to ethnic minorities, lived alone, were current smokers, and were heavy drinkers. Males also had a higher BMI and PIR and were more likely to participate in vigorous PA. Additionally, the rate of diabetes and CVD were higher, but the rate of CKD was lower in males. There was no significant difference between females and males regarding education level and hypertension prevalence.

### 3.2 Univariate analysis of SαKl levels

Univariate analysis revealed that SαKl levels varied significantly with age, gender, NLR, PLR, race/ethnicity, BMI, smoking status, alcohol consumption, hypertension, CKD, and CVD (all *P* < 0.05, Table S1).

### 3.3 Association between NLR, PLR, and SαKl levels

Three weighted linear regression models were fitted to explore the correlation between NLR, PLR, and SαKl, as shown in Table 2. Through weighted multivariate linear analysis, we observed that both the NLR and PLR were negatively associated with SαKl levels across all three models tested. This negative correlation persisted regardless of whether NLR and PLR were treated as continuous or categorical variables.

**Table 2:** Associations between neutrophil-to-lymphocyte ratio, platelet-to-lymphocyte ratio, and serum α − klotho level in adults aged 40 and above from NHANES 2007–2016 using multivariable linear regression.

	Model 1		Model 2		Model 3	
	β (95 % CI)	*P*	β (95 % CI)	*P*	β (95 % CI)	*P*
**NLR, continuous**	−14.76 (−21.89, −7.64)	<0.001	−9.96 (−16.88, −3.05)	0.006	−8.80 (−15.25, −2.36)	0.010
**NLR, categories**						
Q1	Ref.		Ref.		Ref.	
Q2	–22.44 (−48.07, 3.18)	0.090	−14.08 (−40.89, 12.73)	0.307	−12.64 (−39.42, 14.15)	0.359
Q3	−50.03 (−74.16, −25.90)	<0.001	−39.51 (−64.19, −14.83)	0.002	−37.56 (−61.57, −13.54)	0.003
Q4	−54.13 (−79.43, −28.83)	<0.001	−38.20 (−64.02, −12.38)	0.005	–33.38 (−57.53, −9.22)	0.009
*P* for trend		<0.001		<0.001		<0.001

**PLR, continuous**	−0.29 (−0.45, −0.14)	<0.001	−0.30 (−0.46, −0.14)	<0.001	−0.37 (−0.53, −0.22)	<0.001
**PLR, categories**						
Q1	Ref.		Ref.		Ref.	
Q2	1.40 (–23.98, 26.79)	0.914	2.64 (–22.50, 27.78)	0.838	−2.00 (−27.35, 23.34)	0.878
Q3	−29.48 (−54.27, −4.69)	0.022	−30.93 (−55.43, −6.43)	0.016	−40.57 (−65.94, −15.21)	0.003
Q4	−28.66 (−52.16, −5.16)	0.019	−29.61 (−52.90, −6.31)	0.015	−40.88 (−63.82, −17.93)	<0.001
*P* for trend		0.002		0.001		<0.001

Note: NLR, Q1:0.009–1.464; Q2:1.466–1.944; Q3:1.947–2.565; and Q4:2.571–28.655. PLR, Q1:3.821–92.258; Q2:92.272–116.400; Q3:116.428–147.500; and Q4:147.619–830.000.

Model 1: Non-adjusted.

Model 2: Adjusted for age, gender, race/ethnicity.

Model 3: Adjusted for age, gender, race/ethnicity, BMI, marital status, education level, PIR, smoking status, alcohol consumption, physical activity, hypertension, diabetes, CVD, and CKD.

Abbreviations: CI, Confidence interval; NLR, Neutrophil-to-lymphocyte ratio; PLR, Platelet-to-lymphocyte ratio; BMI, body mass index; PIR, Ratio of family income to poverty; CVD, cardiovascular disease; CKD, chronic kidney disease.

**Fig. 2:**
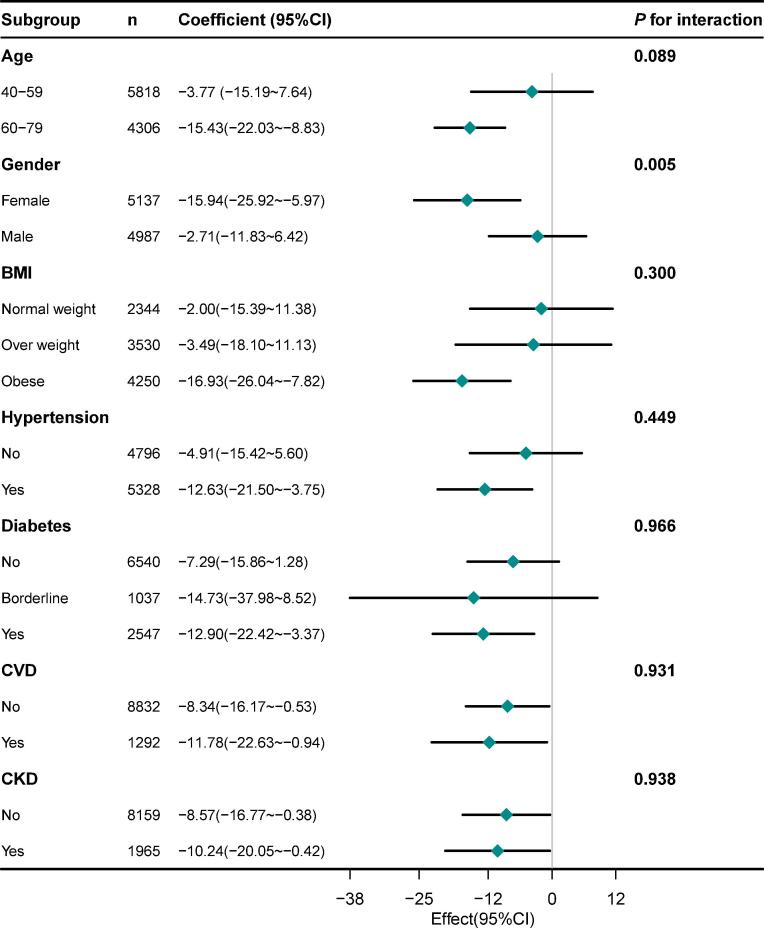
Subgroup analyses of the correlation between neutrophil-to-lymphocyte ratio and serum α-klotho level in adults aged 40 and above, NHANES 2007–2016. Adjusted for age, gender, race/ethnicity, BMI, marital status, education level, PIR, smoking status, alcohol consumption, physical activity, hypertension, diabetes, CVD, and CKD. Abbreviations: BMI, body mass index; PIR, Ratio of family income to poverty; CVD, cardiovascular disease; CKD, chronic kidney disease.

**Fig. 3:**
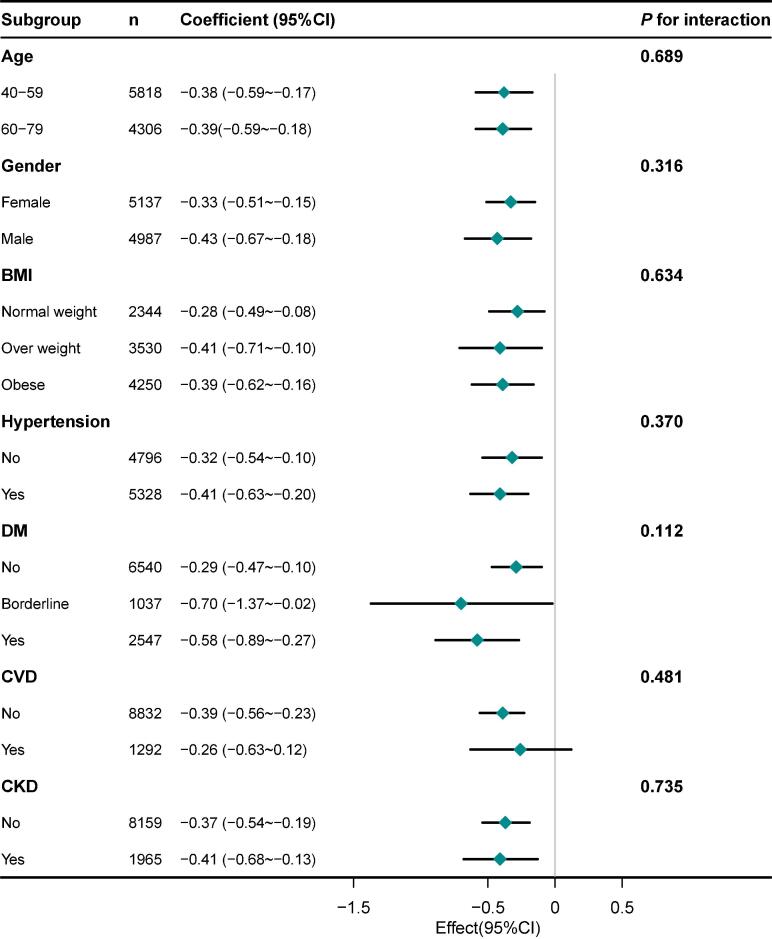
Subgroup analyses of the correlation between platelet-to-lymphocyte ratio and serum α-klotho level in adults aged 40 and above, NHANES 2007–2016. Adjusted for age, gender, race/ethnicity, BMI, marital status, education level, PIR, smoking status, alcohol consumption, physical activity, hypertension, diabetes, CVD, and CKD. Abbreviations: BMI, body mass index; PIR, Ratio of family income to poverty; CVD, cardiovascular disease; CKD, chronic kidney disease.

In the subgroup analysis, we observed that the relationship between NLR, PLR, and SαKl levels in various inflammation-related diseases, including hypertension, diabetes, CVD, and CKD (Fig. 2 and Fig. 3). In individuals with CVD, the negative correlation between PLR and αKl was not significant. The correlation between NLR and SαKl was not significant in participants with no history of hypertension or diabetes. However, in patients with hypertension, diabetes, or CKD, negative correlations were investigated between NLR and SαKl levels. Similarly, the PLR and SαKl levels were also negatively correlated. These results were both of significance which were consistent with the result in the overall population.

Additionally, we analyzed the influence of demographic factors on the relationship between NLR and SαKl levels as well as PLR and SαKl levels. We found that the negative correlation between NLR and SαKl was not significant in individuals aged 40–59 years and males.

## 4 Discussion

The present study investigated the relationship between NLR, PLR, and SαKl levels using data from the NHANES. Our findings indicated a significant negative correlation between both NLR and PLR with SαKl levels in the overall population. Notably, some heterogeneity was observed in the relationship between NLR and SαKl levels across age, gender, hypertension, and diabetes groups. Similarly, variability of PLR and SαKl levels was particularly evident in CVD groups. The findings of this study contributed to the growing evidence supporting the involvement of SαKl in immune and inflammatory processes. However, further research is needed to reveal the underlying mechanism of different SαKl expression in these diseases.

The NLR and PLR derived from routine blood tests emerged as important predictors. Elevated NLR and PLR have been linked to various inflammatory conditions, including infections (Russell et al., 2019), CVD (Haybar et al., 2019), and cancers (Diem et al., 2017, Nost et al., 2021). The present study found that NLR and PLR were significantly negative correlated with SαKl, respectively. SαKl levels were decreased in chronic inflammation status (Kuro, 2019). Elevated neutrophils and platelets, as indicated by increased NLR and PLR, can exacerbate this oxidative stress, leading to a more pronounced reduction in αKl levels (Kuro, 2019). Several mechanisms may account for the negative correlation between NLR, PLR and SαKl levels found in our study: 1) Inflammatory Cytokine Regulation: Inflammatory cytokines (interleukin 6, Tumor Necrosis Factor-α) have been implicated in the downregulation of αKl expression (Moreno et al., 2011, Zhang et al., 2022). 2). Oxidative Stress: Increased oxidative stress may reduce the stability and activity of αKl, contributing to lower αKl levels in inflammatory conditions (Xu and Sun, 2015). 3). Endothelial Dysfunction: Normal αKl production by endothelial cells may disrupt in the inflammatory status, which is the instant cause of the lower αKl level in chronic inflammatory disease (Moreno et al., 2011). 4). Renal Damage: Inflammation may reduce expression and secretion of αKl which is predominantly expressed in the kidneys and plays a crucial role in renal function. Thus, systemic decreases in SαKl levels happened (Prud'homme et al., 2022). 5. Feedback Loop: As an anti-inflammatory protein, decreased αKl levels in inflammatory conditions may exacerbate the inflammatory response and trigger a feedback loop perpetuating the negative association between inflammation and αKl (Izquierdo et al., 2012).

In our subgroup analysis, we examined that the relationship between NLR, PLR, and SαKl levels in various conditions known to involve inflammatory responses, including hypertension, diabetes, CVD, and CKD. In individuals with CVD, the negative correlation between PLR and SαKl was not significant, suggesting variability in the inflammation and SαKl relationship within this group. However, in other subgroups such as hypertension, diabetes, and CKD, the relationship between NLR or PLR with SαKl showed a stronger negative relationship between inflammation and reduced αKl expression.

The lack of a significant relationship between NLR and SαKl levels in males may be attributed to the modulatory effects of sex hormones. Higher levels of testosterone in males exert anti-inflammatory effects (Buendia-Gonzalez and Legorreta-Herrera, 2022), which can influence the relationship between NLR and SαKl levels. Testosterone suppresses specific pro-inflammatory pathways (Mohamad et al., 2019), such as the phospholipase D pathway, leading to reduced leukotriene biosynthesis and decreased inflammation (Pergola et al., 2011). This mitigation of systemic inflammation by testosterone might diminish its impact on αKl expression, explaining the weaker association between NLR and SαKl levels in males. On the other hand, lower levels of systemic inflammation in the middle-aged group than in the older age groups (Singh et al., 2024) might be account for the absence of significant correlation between NLR and SαKl levels in individuals aged 40–59. As people grow older, the severity of chronic inflammation increases, which may lead to a higher prevalence of chronic inflammatory conditions (Walker et al., 2022).

In our study, the relationship between PLR and SαKl levels may be more significant and robust than that between NLR and SαKl levels. Platelets play a crucial role in inflammation by interacting with leukocytes and endothelial cells, releasing pro-inflammatory cytokines and growth factors directly influencing αKl expression (Maouia et al., 2020). PLR and NLR are both calculated by lymphocyte count while PLR may reflect inflammatory as well as the thrombotic activity for the special function of platelets which provide an additional dimension than NLR (Xu and Sun, 2015). Elevated platelet activation and aggregation also contribute to endothelial injury due to inflammation. In summary, the stronger association between PLR and αKl levels may be due to the unique role of platelets in inflammation and its influence on αKl expression.

The strengths of this study include the large sample size and the use of a representative multiethnic population from 5 consecutive NHANES cycles. However, there are several limitations to consider. First, despite adjusting for numerous potential confounders, the study design cannot completely rule out the possibility of residual confounding. Second, we can only observe the correlation between NLR or PLR and αKl but not affirm the causal relationship in an observational study. Third, the use of anti-inflammatory drugs was not included in our study, which may influence our results.

## 5 Conclusion

In conclusion, our study demonstrates a significant negative correlation between inflammatory markers (NLR and PLR) and SαKl levels, emphasizing the role of systemic inflammation in influencing SαKl. Subgroup analyses showed that the relationship varies across different demographic and health-related factors. These findings suggest potential pathways for intervention aimed at reducing inflammation to preserve SαKl levels and promote healthy aging. Further research is needed to explore the underlying mechanisms and validate these findings in diverse populations and clinical settings.

## Ethics approval

The ethical approval to conduct the NHANES was granted by the NHANES Institutional Review Board. The study procedures were structured in line with the Declaration of Helsinki. All participants provided their written informed consent.

## Funding

This study did not receive any specific grant from funding agencies in the public, commercial, or not-for-profit sectors.

## CRediT authorship contribution statement

**Rui Du:** Writing – review & editing, Writing – original draft, Supervision, Software, Methodology, Investigation, Conceptualization. **Jie Liu:** Writing – original draft, Formal analysis, Conceptualization. **Xiaoyan Tang:** Software, Methodology. **Zili Chen:** Methodology, Investigation, Formal analysis. **Lei Guan:** Software, Investigation. **WenHong Gao:** Software. **Wei Huang:** Writing – review & editing, Writing – original draft, Supervision, Project administration, Methodology, Investigation, Formal analysis, Conceptualization.

## Declaration of competing interest

The authors declare that they have no known competing financial interests or personal relationships that could have appeared to influence the work reported in this paper.

## Data Availability

The datasets used and/or analysed during the current study are available from the corresponding author on reasonable request.
