# Supplementary material for: Correlation of neutrophil-to-lymphocyte ratio and platelet-to-lymphocyte ratio with serum α-klotho levels in US middle-aged and older individuals: Results from NHANES 2007–2016
Source: Prev Med Rep. 2024 Sep 6;46:102877. doi: 10.1016/j.pmedr.2024.102877 (PMC11415581; doi:10.1016/j.pmedr.2024.102877)
Supplement: Supplementary Data 1 [file mmc1.docx]

**Table S1. Univariate analysis for α-klotho level (pg/mL)** **in adults aged 40 and above from NHANES 2007-2016**

|  | **N** | **Mean (95%CI)** | **Coeff. (95%CI)** | ***P*** |
| --- | --- | --- | --- | --- |
| **NLR, continuous** | 10124 | 848.94 (838.39, 859.48) | -14.76 (-21.89, -7.64) | <0.001 |
| **NLR4, categories** |  |  |  |  |
| Q1 | 2525 | 882.80 (862.24, 903.36) | Ref. |  |
| Q2 | 2536 | 860.36 (840.69, 880.03) | -22.44 (-48.07, 3.18) | 0.090 |
| Q3 | 2524 | 832.76 (818.24, 847.29) | -50.03 (-74.16, -25.90) | <0.001 |
| Q4 | 2539 | 828.66 (813.36, 843.96) | -54.13 (-79.43, -28.83) | <0.001 |
| **PLR, continuous** | 10124 | 848.94 (838.39, 859.48) | -0.29 (-0.45, -0.14) | <0.001 |
| **PLR4, categories** |  |  |  |  |
| Q1 | 2531 | 864.03 (843.80, 884.26) | Ref. |  |
| Q2 | 2529 | 865.43 (848.72, 882.15) | 1.40 (-23.98, 26.79) | 0.914 |
| Q3 | 2529 | 834.55 (818.14, 850.96) | -29.48 (-54.27, -4.69) | 0.022 |
| Q4 | 2535 | 835.37 (819.04, 851.70) | -28.66 (-52.16, -5.16) | 0.019 |
| **Age** | 10124 | 848.94 (838.39, 859.48) | -1.88 (-2.56, -1.20) | <0.001 |
| **Gender** |  |  |  |  |
| Female | 5137 | 867.92 (854.92, 880.93) | Ref. |  |
| Male | 4987 | 828.90 (816.52, 841.28) | -39.02 (-53.07, -24.98) | <0.001 |
| **Race/ethnicity** |  |  |  |  |
| Mexican American | 1662 | 852.77 (833.73, 871.81) | Ref. |  |
| Other Hispanic | 1146 | 874.00 (847.38, 900.63) | 21.23 (-9.54, 52.00) | 0.180 |
| Non-Hispanic White | 4304 | 839.15 (827.54, 850.76) | -13.63 (-35.23, 7.97) | 0.220 |
| Non-Hispanic Black | 2063 | 909.85 (882.88, 936.82) | 57.08 (25.24, 88.92) | <0.001 |
| Others | 949 | 848.40 (820.01, 876.79) | -4.37 (-35.33, 26.59) | 0.783 |
| **BMI** |  |  |  |  |
| Normal weight | 2344 | 874.01 (854.92 ,893.10) | Ref. |  |
| Over weight | 3530 | 842.20 (825.52 ,858.88) | -31.81 (-55.83, -7.78) | 0.011 |
| Obese | 4250 | 840.02 (827.78 ,852.25) | -33.99 (-55.05, -12.93) | 0.002 |
| **Marital status** |  |  |  |  |
| Married/living with partner | 6570 | 844.68 (833.13, 856.23) | Ref. |  |
| Living alone | 3554 | 859.16 (843.13, 875.19) | 14.48 (-2.45, 31.42) | 0.098 |
| **PIR** |  |  |  |  |
| Low | 3084 | 844.71 (829.29 ,860.14) | Ref. |  |
| Middle | 3077 | 849.16 (832.00 ,866.32) | 4.45 (-15.91, 24.80) | 0.669 |
| High | 3963 | 850.17 (836.83 ,863.50) | 5.45 (-13.63, 24.53) | 0.577 |
| **Education level** |  |  |  |  |
| Less than high school | 2772 | 844.66 (825.21, 864.12) | Ref. |  |
| High school or GED | 2275 | 827.12 (812.19, 842.06) | -17.54 (-41.02, 5.95) | 0.147 |
| Above high school | 5077 | 858.28 (843.95, 872.60) | 13.61 (-8.33, 35.56) | 0.228 |
| **Smoking status** |  |  |  |  |
| Never smoker | 5211 | 864.02 (849.94, 878.10) | Ref. |  |
| Former smoker | 2888 | 840.45 (825.42, 855.48) | -23.57 (-40.95, -6.19) | 0.010 |
| Current smoker | 2025 | 819.87 (801.93, 837.82) | -44.15 (-64.52, -23.77) | <0.001 |
| **Alcohol consumption** |  |  |  |  |
| Never drinker | 1461 | 888.38 (864.34, 912.42) | Ref. |  |
| Former drinker | 2164 | 856.60 (840.13, 873.07) | -31.78 (-59.87, -3.70) | 0.030 |
| Light to moderate drinker | 4872 | 852.39 (839.15, 865.63) | -35.99 (-60.70, -11.28) | 0.006 |
| Heavy drinker | 1627 | 803.67 (784.53, 822.81) | -84.71 (-114.72, -54.71) | <0.001 |
| **Physical activity** |  |  |  |  |
| Inactive | 5686 | 849.42 (836.75, 862.08) | Ref. |  |
| Moderate | 2853 | 835.30 (820.11, 850.50) | -14.11 (-31.48, 3.25) | 0.115 |
| Vigorous | 1585 | 869.22 (846.46, 891.98) | 19.80 (-3.51, 43.12) | 0.100 |
| **Hypertension** |  |  |  |  |
| No | 4796 | 857.27 (845.43, 869.12) | Ref. |  |
| Yes | 5328 | 839.55 (826.21, 852.89) | -17.72 (-31.46, -3.98) | 0.014 |
| **Diabetes** |  |  |  |  |
| No | 6540 | 849.25 (837.81, 860.68) | Ref. |  |
| Borderline | 1037 | 841.12 (815.66, 866.58) | -8.13 (-33.97, 17.71) | 0.539 |
| Yes | 2547 | 852.04 (835.00, 869.09) | 2.80 (-14.04, 19.63) | 0.746 |
| **CVD** |  |  |  |  |
| No | 8832 | 853.77 (842.84, 864.69) | Ref. |  |
| Yes | 1292 | 806.28 (787.20, 825.36) | -47.49 (-66.32, -28.65) | <0.001 |
| **CKD** |  |  |  |  |
| No | 8159 | 854.96 (843.11, 866.81) | Ref. |  |
| Yes | 1965 | 815.67 (798.90, 832.45) | -39.29 (-59.07, -19.51) | <0.001 |

Note: NLR, Q1:0.009-1.464; Q2:1.466-1.944; Q3:1.947-2.565; and Q4:2.571-28.655. PLR, Q1: 3.821-92.258; Q2:92.272-116.400; Q3:116.428-147.500; and Q4:147.619-830.000.

**Abbreviations:** NLR, Neutrophil-to-lymphocyte ratio; PLR, Platelet-to-lymphocyte ratio; BMI, body mass index; PIR, Ratio of family income to poverty; CVD, cardiovascular disease; CKD, chronic kidney disease.
